# Epidemiological and clinical aspects of ulcerative colitis in west of Iran: a cross sectional study

**DOI:** 10.1186/s40064-016-3248-4

**Published:** 2016-09-15

**Authors:** Koroush Ghanadi, Javad Valizadeh, Afshin Hasanvand

**Affiliations:** 1 Gastroenterology and Hepatology, Lorestan University of Medical Sciences, Khorramabad, Iran; 2 Student Research Committee, Lorestan University of Medical Sciences, Khorramabad, Iran

**Keywords:** Ulcerative colitis, Inflammatory bowel disease (IBD), Clinical characteristics, Iran

## Abstract

**Introduction:**

One of the forms of inflammatory bowel disease (IBD) that causes inflammation and ulcers in colon is ulcerative colitis (UC). This study was aimed to determine the epidemiological and clinical aspects of patients with ulcerative colitis in the west of Iran.

**Methods:**

In this cross sectional study, we evaluated 150 patients with definite diagnosis of UC who referred to a subspecialty gastroenterology clinic in Khorramabad, Iran. The study was performed from May 2014 to August 2015 by using census method. Demographic characteristics as well as patients’ clinical profiles were extracted using a checklist. Disease severity was determined by the Truelove and Witt’s classification of ulcerative colitis (UC). Data were analyzed using SPSS software, version 17 for windows (IBM Inc., NY, US).

**Results:**

A total of 150 patients with definite diagnosis of UC were analyzed, including 84 (56 %) women and 66 (44 %) men (male/female ratio = 1.27). The mean age of patients was 33.7 ± 12.5 years with an age range of 17–98 years. The age of the majority of patients was 20–29 and most of them (56 %) were women and living in urban areas (70.7 %). The most common site of the involvement in colon was rectosigmoid (66 patients, 44 %). Severity of the disease was mild in 36 patients (24.1 %), moderate and severe in 74 and 40 patients (49.3 and 26.6 %), respectively. The most common clinical manifestation of the patients was dysentery (68 %) and then rectorrhagia (60 %). Only 12 of all patients (7.8 %) mentioned a positive family history of IBD in their first degree kinfolks. Ten patients (6.7 %) had a history of regular smoking and four of them (2.6 %) mentioned a history of appendectomy. Four patients (5.3 %) had a positive history of smoking by their mothers during their pregnancy.

**Conclusion:**

The results of this study demonstrate differences and similarities in demographic and clinical characteristics of UC in this part of Iran in comparison to other parts of the country.

## Background

Inflammatory bowel disease (IBD) is known as a group of inflammatory conditions (signs and symptoms) of the colon and small intestine. Ulcerative colitis (UC) and Crohn’s disease (CD) are the most important types of IBDs. IBD causes the bowel (intestine) to be inflamed, swollen, irritated and unable to function in a healthy way. UC is a form of IBDs that leads to inflammatory and ulcerative lesions in the colon. It affects large intestine (the colon) and rectum but does not involve the small intestine; whereas CD affects both the colon and small intestine (Torpy et al. 2012; Danese and Fiocchi 2011). Although considerable progresses have been made in IBD research, but researchers are yet to determine the exact etiology of the disease. Some studies suggested that this disease involves a complex interaction of factors such as a variety of genetic, immunologic and environmental factors (Shirazi et al. 2013a) (4 Website). Diarrhea mixed with blood is the main symptom of the active form of the disease and the other symptoms are usual such as mucous stool, abdominal pain and extra-intestinal manifestations (Torpy et al. 2012; Li et al. 2015). UC is characterized by recurring episodes of inflammation limited to the mucosal layer of the colon. It is a relapsing and remitting disorder with disease free intervals (Thoreson and Cullen 2007). The incidence and prevalence of UC depend on geographic location and ethnicity. UC has an incidence rate of 1–20 cases per 100,000 individuals/year, and a prevalence of 8–246/100,000 individuals (Danese and Fiocchi 2011). IBD is reportedly more common in developed countries than developing countries. However, according to some recent studies, the prevalence of IBD is on the increase in developing nations in the past two decades (Thia et al. 2008; Balaii et al. 2015). The total number of new cases of UC each year in North America is 10–12/100,000 individuals/year. It is common in people between the ages of 15 and 25. The number of affected individuals is 1–3/1000 (Busch et al. 2014). The disease affects females more than males (Hanauer 1996). The geographic distribution of UC and CD is similar worldwide (Podolsky 2002) with the highest incidences in the United States, Canada, the United Kingdom, and Scandinavia. Higher incidences are seen in northern climates as compared with southern climates in Europe (Shivananda et al. 1996) and the United States (Sonnenberg et al. 1991). Studies of Asians in Asia show relatively low incidence rates for UC and CD as compared with North America and Europe. The prevalence of both UC and CD in Japan and Korea is relatively low (Yang et al. 2001). Although the evidence is insufficient, there are reports of the growing incidence of UC in the Middle East (Siddique et al. 2012; Abdul-Baki et al. 2007a, b; Aghazadeh et al. 2005a). Recently, the incidence rate in Arab population was reported to be 22/100,000 (El Mouzan et al. 2006; Lennard-Jones 1989; Khan et al. 1996; Contractor et al. 2004). Moreover, a recent retrospective study in Saudi Arabia reported an increase in the number of UC patients who were referred to tertiary care centers (Alamin et al. 2001; Alharbi et al. 2014). Another retrospective study on Libyan children showed that the incidence of IBD was increasing and the clinical features were similar to those reported in other countries (Ahmaida and Al-Shaikhi 2009). Similar to other parts of Asia, previous reports from Iran have demonstrated an increasing incidence of IBD, particularly UC, in this region (Mir-Madjlessi et al. 1985; Vahedi et al. 2009b; Aghazadeh et al. 2005a; Daryani et al. 2006). Certain lifestyle and socioeconomic conditions in combination with Genetic and Immunologic factors seem to be involved in the development of UC. In addition, a familial incidence of UC has been recognized for many years, and about 10–20 % of patients have at least one other family member that is affected by UC. A number of environmental risk factors have been proposed to be associated with UC, such as diet, food additives, Appendectomy, oral contraceptive, cigarette smoking, alcohol abuse and so forth. Some studies have indicated that cigarette smoking and Appendectomy may have a protective role against the development of UC (López-Serrano et al. 2010). Despite previous reports from Iran have suggested increasing incidence of UC, there is limited data regarding the characteristics of these patients and disease course in Iran. This study was conducted to determine the epidemiological and clinical characteristics of UC in Khorramabad, Iran, from May 2014 to August 2015.

## Methods

The present study was performed from May 2014 to August 2015 using census method. A total of 150 patients with UC, who were referred to a subspecialty clinic of gastroenterology sequentially in Khorramabad, Iran were included in this study. They were diagnosed of UC based on clinical manifestations, colonoscopy, stool examination and pathologic confirmation. In order to prevent breaches of patient’s privacy, clinical history and laboratory evaluations were recorded with no name or telephone number.

### Ethical aspects

The study protocol was in accordance with the Declaration of Helsinki for investigation involving human subjects and was approved by the ethics committee of Lorestan University of Medical Sciences, Khorramabad, Iran.

### Exclusion criteria

Patients with incomplete data were excluded from the study.

### Data collection

To gather information about the patients, personal interview was carried out by a gastroenterologist and the obtained information was recorded in a questionnaire. The patients’ age, sex, clinical symptoms, disease duration, history of smoking, oral contraceptive use, appendectomy and a positive family history of IBD in their first degree kinfolks were recorded in the first part of the questionnaire. The second part of the questionnaire included information about clinical aspects of the disease such as intestinal and extra intestinal manifestations, disease severity, the site of colon involvement based on colonoscopic findings and kind of drugs used. To evaluate some of the extra intestinal manifestations in addition to history taking and physical examination, some of the laboratory tests, and liver, gall bladder, kidney and urinary tract sonography were requested for all the patients. Disease severity was determined using the Truelove and Witt’s classification of UC.

### Statistical analysis

 Data were analyzed using SPSS software, version 17 for windows (IBM Inc., NY, US). Descriptive tests were used as appropriated.

## Results

In this study, a total of 150 patients diagnosed with UC were evaluated. Out of the 150 patients examined, 84 (56 %) were women and 66 (44 %) were men (male/female ratio = 1.27). The mean age of the patients was 33.7 ± 12.5 years with an age range of 17–98 years. The age range of majority of the patients in this study was 20–29 (38.7 %). Most of them (56 %) were women and living in urban areas (70.7 %). The educational level of most of them was high school and Diploma (48 %). The frequency distribution of the demographic characteristics of the patients is shown in Table 1. The mean age at disease onset was 29.4 ± 12.1 years and the mean duration from the first symptom of the disease to the definite diagnosis was 18.9 months. Relapse occurred in 50 patients (33.3 %). Based on colonoscopic findings, the most common site of colon involvement was rectosigmoid (66 patients, 44 %). Rectum involvement was seen in 38 patients (25.3 %), left colon and pan colon involvement were seen, respectively in 26 and 20 patients (17.3 and 13.3 %). In terms of severity of the disease based on colonoscopy, disease severity was mild in 36 patients (24.1 %), moderate and severe, respectively in 74 and 40 patients (49.3 and 26.6 %). The most common clinical manifestation of the patients in this study was dysentery (68 %), followed by rectorrhagia (60 %). The frequency distribution of clinical signs and symptoms of the disease is shown in Table 2. Extra intestinal manifestations were seen in 22 patients (14.3 %) of which the most common of them were aphthous ulcers with 2.6 % each. Hepatobilliary symptoms were the most common extra intestinal manifestation with 6.7 % (Table 3). In terms of the kind of treatment, majority of the patients (21.3 %) were treated with mesalamine and asacol Suppositories. Thus 18.7, 13.3 and 17.3 % of the patients took only asacol, combination of corticosteroids, mesalamine and asacol and combination of corticosteroids and mesalamine, respectively. Four patients (2.6 %) required surgery. Only 12 among all patients (7.8 %) mentioned a positive family history of IBD in their first degree kinfolks. Ten patients (6.7 %) had a history of regular smoking and four of them (2.6 %) mentioned a history of appendectomy. Majority of the patients (37.3 %) were second child in their family and the mean age of their mothers as at the time of their pregnancy was 29.1 years. Four patients (5.3 %) had a positive history of smoking by their mothers during their pregnancy. With regards to delivery type, 24 % of the patients were through C/S and only 66 patients (44 %) were breastfed and the other patients were fed by formulas and other milks (Table 4).

**Table 1:** frequency distribution of individual characteristics of the patients with IBD

Variable	Absolute frequency (number)	Relative frequency (percent)	Additive frequency (percent)
Age			
<20	12	(8)	(8)
20–29	58	(38.7)	(46.7)
30–39	34	(22.7)	(69.3)
40–49	30	(20)	(89.3)
≥50	16	(10.7)	(100)
Sex			
Male	66	(44)	(44)
Female	84	(56)	(100)
Marriage status			
Single	104	(69.3)	(69.3)
Married	42	(28)	(97.3)
Others	4	(2.7)	(100)
Literacy			
Uneducated	16	(10.7)	(10.7)
Primary and secondary	30	(20)	(30.7)
High school	72	(48)	(78.7)
Academic	32	(21.3)	(100)
Job			
Unemployed	12	(8)	(8)
Worker	4	(2.7)	(10.7)
Clerk	6	(4)	(14.7)
Farmer	8	(5.3)	(19.7)
Housewife	70	(46.7)	(66.4)
Free	46	(30.7)	(97.3)
Military	4	(2.7)	(100)
Residency			
Urban	106	(70.7)	(70.7)
Rural	44	(29.3)	(100)

**Table 2:** Frequency distribution of clinical manifestations of ulcerative colitis in studied patients

Sign or symptom	Absolute frequency (number)	Relative frequency (percent)
Rectorrhagia	(90)	(60)
Diarrhea	(40)	(26.7)
Dysentery	(102)	(68)
Constipation	(22)	(14.7)
Abdominal pain	(56)	(37.3)
Defecation with pain	(82)	(54.7)
Incomplete defecation	(62)	(41.3)
Vomiting	(12)	(8)
Weight loss	(78)	(52)
Melena	(6)	(4)
Anemia	(40)	(26.7)
Anal fistula	(10)	(6.7)
Fever	(12)	(8)
Weakness/fatigue	(60)	(40)

**Table 3:** Extra intestinal manifestations of IBD in studied patients

Extra intestinal manifestation	Absolute frequency (number)	Relative frequency (percent)
Kidney stone	(2)	(1.3)
Gall bladder stone	(2)	(1.3)
Arthritis	(2)	(1.3)
Erythema nodosum	(2)	(1.3)
Primary sclerosing cholangitis	(2)	(1.3)
Deep vein thrombosis	(2)	(1.3)
Aphthous ulcers	(4)	(2.6)
Fatty liver	(2)	(1.3)

**Table 4:** Frequency of involved segments of colon

Involved segment	Absolute frequency (number)	Relative frequency (percent)
Rectum	38	(25.3)
Rectosigmoid	66	(44)
Left colon	26	(17.3)
Pan colon	20	(13.3)

## Discussion

UC is a chronic disease of the colon (also known as the large intestine) associated with inflammation of the lining of colon and development of tiny open sores, or ulcers, which produce mucous and pus. The combination of ulceration and inflammation can cause abdominal discomfort and also frequent emptying of the colon. The disease is the result of an abnormal response by the immune system of the body. The cells and proteins which make up the immune system, normally protect the individual from infections. However, in people with IBD, food, bacteria and other materials are mistaken for antigen by the immune system. Hence, the body sends white blood cells into the lining of the intestines and cause chronic inflammation and ulcerations. The present study was conducted to assess the clinic-epidemiological characteristics and colonoscopic findings of UC in Khorramabad city from May 2014 to August 2015. UC most commonly begins between the ages of 15 and 25. Also, another usual age of the onset is in the 6th decade of life. Females are more likely to be affected than males (Büsch et al. 2014). In this study, the mean age of the patients was 33.7 ± 12.5 years with an age range of 17 to 98 years. The percentage of affected women with a shade was higher than men, and this is in line with the results of several studies conducted in Iran and across the globe (male/female ratio = 1.27). In a study on the demographic and clinical features of UC patients in Kerman, the mean age of the patients was reported as 33.3 years and the sexual proportion of males to females was 0.8–1 (Zahedi et al. 2009). In another study, the mean age of the patients was 32 years and the percentage of affected women with a shade was higher than men (Abdul-Baki et al. 2007a, b). Keshavarz and Izadi (2008) found the mean age of the patients to be 34 years and the sexual proportion of males to females as 1.08, indicating that the percentage of the men with a shade was higher than women in this study. The disparity in findings between the present study and other studies can be attributed to the difference in sample content of the studies and number of patients used. The mean age at onset of the disease in this study was 29.4 years and the mean duration from the first symptom of the disease to the definite diagnosis was 18.9 months. The long duration from symptomatic to definite diagnosis in the present study may be attributed to lack of gastroenterologists in the province, and the referring of patients to general physicians’ clinics and delay in diagnosis. In the present study, relapse occurred in 33.3 % of the patients. In most cases, improper attention to the given orders and irregular drug use by the patients were the reasons for the frequent disease relapse. The most and least common site of colon involvement in this study were rectosigmoid and pan colon, respectively. This finding is consistent with the results of previous studies (Aghazadeh et al. 2005b; Farmer et al. 1993; Keshavarz and Izadi 2008). In a study Portela et al. (2010) in southern European countries, it was reported that the most common site of involvement was the left colon. Pan colon involvement was seen in 28 % of cases in this study. With regards to the site of colon involvement, there is a relative similarity between the results of the present study and other studies. The reason for the difference in site of colon involvement may be due to the difference in disease pathogenesis in different places in the world or could be attributed to the fact that colonoscopy is performed by different persons in world and that sometimes, the gastroenterologist may not have performed this test to determine the exact length of the involved colon. In a study conducted in China on 389 patients with UC, the disease severity was recorded as moderate and severe in 52.4 and 13.4 % of the patients, respectively (Jiang et al. 2006), and this is consistent with the results of the present study, as the disease severity of 49.3 % of patients was considered as moderate. In the present study, the most common clinical manifestations seen in patients were dysentery and rectorrhagia. In a study conducted on Epidemiological and clinical characteristics of IBD in patients from northwestern Iran, it was shown that the most common clinical manifestations of patients with UC were abdominal cramp and rectorrhagia (Shirazi et al. 2013b), whereas in the present study, the most common clinical manifestations were dysentery and rectorrhagia. The most common extra intestinal manifestations in the present study were aphthous ulcers, but in general, hepatobiliary manifestations were the most common form of extra intestinal involvement in this study. Notably, 14.3 % of our patients had extra intestinal manifestations. Aghazadeh et al. (2005b) and Vahedi et al. (2009a) reported that 31.4 and 62.4 % of their patients had extra intestinal manifestations, respectively. While in a study by Jiang and Cui (2002), the frequency of extra intestinal manifestations was determined as 6.1 %. The disparity in findings between the present study and other studies can be attributed to fewer number of sample content in the present study and the inability to evaluate all the extra intestinal manifestations in the patients. With regards to family history, in the present study, the family history of IBD in the first degree kinfolks of the patients was 7.8 %. In a study conducted by Bahari et al. (2004), 6.5 % of the patients had a positive history of smoking, which is consistent with the results of the present study. In study byYazdanbod et al. (2011), the family history of IBD in the first degree kinfolks of the patients were 10.9 and 5.4 %, respectively. De Saussure et al. (2007), in their study, reported a prevalence of appendectomy in patients with UC and control group as 12 and 46 %, respectively demonstrating that appendectomy is a protective factor against UC. Whereas, in the present study, only 2.7 % of patients had a history of appendectomy.

## Conclusion

Ulcerative colitis is one of the important referral causes of patients to the subspecialty clinics of gastroenterology and unofficial records demonstrate increasing incidence of cases of this disease in Iranian race that may be due to changing of people´s life style and following the life style of western communities and developing of diagnostic and treatment instruments of the disease.

Altogether, it seems that the epidemiologic profile and clinical characteristics of UC in our country, Iran, have a relative similarity to other places in the world, but clinical development of the disease is milder and extra intestinal manifestations are fewer; on the other hand, evaluation of all sides of the disease may have an important role to recognize real factors causing the disease and prevent from them. The high rate of frequent relapse of the disease in the patients requires more attention to their treatment and to make them pay attention to the correct and regular medicine taking. Since Khorramabad is the center of Lorestan province in which rather all patients from another parts of the province are referred to there, this city could be representative of population of the west of Iran; in addition there are some other deprived provinces such as Ilam in the west of Iran that the patients of theirs are also referred to Khorramabad to get better services. The results of this study, demonstrate differences and similarities in demographic and clinical profile of UC in this part of Iran in comparison to other parts of the country. Some other limitations of the present study were the short duration of the evaluation of the disease and few numbers of the studied patients.

## Abbreviations

UC: ulcerative colitis
IBD: inflammatory bowel disease
CD: Crohn’s disease
